# Theoretical Insights into the Electron Capture Behavior of H_2_SO_4_···N_2_O Complex: A DFT and Molecular Dynamics Study

**DOI:** 10.3390/molecules23092349

**Published:** 2018-09-13

**Authors:** Wei-Hua Wang, Wen-Ling Feng, Wen-Liang Wang, Ping Li

**Affiliations:** Key Laboratory of Life-Organic Analysis, School of Chemistry and Chemical Engineering, Qufu Normal University, Qufu 273165, China; wlfengqf@163.com (W.-L.F.); wlwangqf@126.com (W.-L.W.)

**Keywords:** sulfuric acid, nitrous oxide, electron capture, theoretical calculations

## Abstract

Both sulfuric acid (H_2_SO_4_) and nitrous oxide (N_2_O) play a central role in the atmospheric chemistry in regulating the global environment and climate changes. In this study, the interaction behavior between H_2_SO_4_ and N_2_O before and after electron capture has been explored using the density functional theory (DFT) method as well as molecular dynamics simulation. The intermolecular interactions have been characterized by atoms in molecules (AIM), natural bond orbital (NBO), and reduced density gradient (RDG) analyses, respectively. It was found that H_2_SO_4_ and N_2_O can form two transient molecular complexes via intermolecular H-bonds within a certain timescale. However, two molecular complexes can be transformed into OH radical, N_2_, and HSO_4_^−^ species upon electron capture, providing an alternative formation source of OH radical in the atmosphere. Expectedly, the present findings not only can provide new insights into the transformation behavior of H_2_SO_4_ and N_2_O, but also can enable us to better understand the potential role of the free electron in driving the proceeding of the relevant reactions in the atmosphere.

## 1. Introduction

As the primary component in the production of secondary aerosols, sulfuric acid (H_2_SO_4_) plays a central role in atmospheric aerosol nucleation [1,2,3,4]. It is mainly produced from sulfur dioxide via oxidation by hydroxyl radical. Generally, when H_2_SO_4_ collides with other molecules, it can form small clusters of molecules that can grow into new stable aerosols [1]. As a result, many interaction models of H_2_SO_4_ with water, ammonia, and organic molecules have been constructed to elucidate new atmospheric particle formation and nucleation mechanisms [5,6,7,8,9,10]. Meanwhile, H_2_SO_4_ also plays an important role in perturbing atmospheric hydroperoxyl radical levels via the formation of strong H-bonding complexes [11,12]. In addition, H_2_SO_4_ has been involved in the formation of the polar stratospheric clouds (PSCs) associated with the stratospheric ozone depletion [13]. Therefore, it is necessary to acquire the detailed interactions of H_2_SO_4_ with other atmospheric species so as to better understand the potential role of H_2_SO_4_ in the atmosphere. 

As one of the important nitrogen-containing species in the atmosphere and a main greenhouse gas, nitrous oxide (N_2_O) has a long atmospheric lifetime and radiative forcing of about 200 times higher than that of CO_2_ [14,15]. It has been demonstrated that the emission of N_2_O is mainly from both natural (about 60%) and anthropogenic sources (about 40%) [16]. In 2016, the globally averaged N_2_O concentration reached 328.9 ± 0.1 ppb, which is 122% of the pre-industrial level (270 ppb) [16]. In addition, N_2_O has also been implicated in the ozone depletion. Ravishankara’s [17] studies suggested that N_2_O emission is the single most important ozone-depleting emission and is expected to remain the largest throughout the 21st century. Therefore, it is also necessary to investigate the transformation of N_2_O to reduce its negative effects on environments.

Given the fact that H_2_SO_4_ and N_2_O coexist in the atmosphere, it is expected that both of them could interact with each other in some way. Can they form stable complexes? If so, what are the interaction modes adopted between them? Especially, what happens to these complexes if an additional electron is introduced since a large number of electrons can be generated by cosmic ray radiation in the atmosphere? Here, it is well known that the excess electrons can be captured by the stratospheric molecules [18,19,20] and the cosmic-ray-driven electron-induced reactions have been involved in the ozone depletion and global climate change [21,22,23]. Unfortunately, despite the atmospheric importance of H_2_SO_4_ and N_2_O, the interaction behavior between them in the absence and presence of an additional electron remains unclear. No relevant studies have been reported so far to the best of our knowledge. In this case, theoretical studies are highly desirable to carry out to provide useful information for the relevant experiments.

Therefore, to address those questions mentioned above, the interaction behavior between H_2_SO_4_ and N_2_O before and after the electron capture has been systematically explored by using the density functional theory (DFT) method and ab initio molecular dynamics simulation. Hopefully, the present study can provide new insights into the potential role of H_2_SO_4_ and the excess electrons in the atmosphere as well as the transformation of N_2_O.

## 2. Computational Details

In this study, full geometric optimizations were performed employing the B3LYP and M06-2X methods as well as the 6-311++G(3df,3pd) basis set, where the reliability of the DFT method has been confirmed by many systems [24,25,26,27,28,29]. As a result, it was found that both methods can give consistent results for the geometry and energy parameters overall. Given the compromise between computational accuracy and cost, and to keep the consistency of the results for the optimization and molecular dynamics at the same level of theory, the results at the B3LYP/6-311++G(3df,3pd) level of theory have been mainly discussed below unless otherwise noted. To explore the nature of the optimized structure, we performed vibration frequency analysis at the same level of theory. Moreover, intrinsic reaction coordinate (IRC) calculations have been performed to further confirm the connectivity between the given transition state and the corresponding reactant and product. In addition, to further improve the energy parameters in the formation process of the neutral complexes, single-point energy calculations have also been carried out at the CCSD(T)/AUG-cc-pVTZ level of theory based on the optimized geometries mentioned above.

To explore the implicit solvent effects on the title reaction, the solvent model density (SMD) solvation model has been employed in aqueous solution at the B3LYP/6-311++G(3df,3pd) level of theory. As a result, similar results have been obtained as those of the results in the gas phase.

To characterize the intermolecular H-bonds between H_2_SO_4_ and N_2_O before and after electron capture, atoms in molecules (AIM) [30], natural bond orbital (NBO) [31], and the reduced density gradient (RDG) analyses have been performed based on the optimized geometries [32,33]. In the AIM theory, the presence and nature of the intermolecular H-bonds can be characterized by the bond critical point (BCP). As for the formation mechanism of the intermolecular H-bonds, it can be elucidated from the orbital interactions between the lone pairs of the proton acceptor and the anti-bonding σ orbital (σ*) of the proton donor within the NBO method, accompanying with the electron transfer from the former to the latter. Moreover, the orbital interaction strength can be assessed from the second-order stabilization energy *E*^(2)^. In addition, the intermolecular H-bonds can also be indicated by the presence of the spikes within the RDG analyses.

To obtain the interaction strength between H_2_SO_4_ and N_2_O, interaction energy between them has been calculated as the energy differences between the formed complexes and the reactants. Meanwhile, zero-point vibrational energy (ZPVE) corrections and basis set superposition errors (BSSEs) have also been considered [34].

To investigate the dynamic stability of the formed complexes and the microscopic details of the electron capture process for those complexes, we performed ab initio molecular dynamics simulation employing the Atom Centered Density Matrix Propagation (ADMP) method at the B3LYP/6-311++G(3df,3pd) level of theory on the basis of their optimized geometries [35,36,37]. The whole simulation was performed at 298 K for the neutral complexes and the electron captured products with a canonical ensemble. For the electron capture processes of the neutral complexes, a microcanonical ensemble has been employed. The total simulation time is 1 ps with a time step of 0.5 fs.

To evaluate the ability to accept an electron for those neutral complexes, the adiabatic electron affinity (AEA), vertical electron affinity (VEA), and vertical electron detachment energy (VEDE) have been calculated. Here, AEA is calculated as the enthalpy difference between the optimized neutral and anionic complexes and VEA is calculated as the electronic energy difference between the optimized neutral and anionic states on the basis of the optimized neutral complexes. As for the VEDE, it is calculated as the electronic energy difference between the neutral and anionic states at the same geometry of the anionic state.

All the calculations were completed employing Gaussian 09 program (Gaussian, Inc., Wallingford, CT, USA) [38].

## 3. Results and Discussion

### 3.1. Formation of Neutral Molecular Complexes

#### 3.1.1. Structural Features

**Figure 1:**
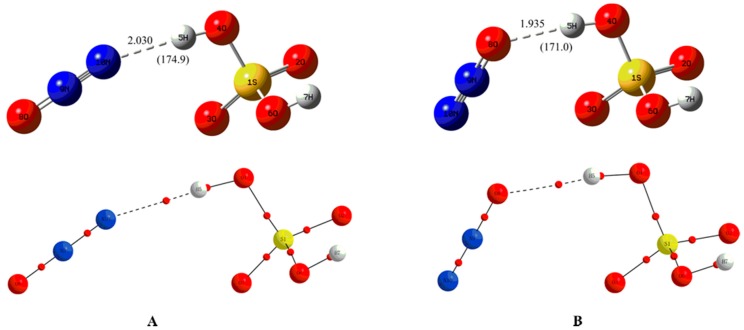
The optimized neutral complexes (**A**) and (**B**) and their molecular graphs, where the units of the bond distances and angles in parentheses are in angstroms and degrees, respectively. The bond critical point (BCP) is denoted as a small red dot.

As displayed in Figure 1, two complexes named as A and B have been formed between H_2_SO_4_ and N_2_O through intermolecular H-bonds. Namely, one of the protons in H_2_SO_4_ interacts with the terminal N and O atoms of N_2_O in complexes A and B, respectively. This point can be confirmed by the location of the corresponding BCPs between H_2_SO_4_ and N_2_O as shown in Figure 1. The intermolecular H-bond distances of 2.030 (2.171) and 1.935 (1.895) A suggest that the H-bonding interactions should be weak, where the data in parentheses refer to the results at the M06-2X/6-311++G(3df,3pd) level of theory. Actually, as shown in Table 1, the positive Laplacians and energy densities at the BCPs of the H-bonds indicate that the above H-bonds are predominated by electrostatic interaction. 

**Figure 2:**
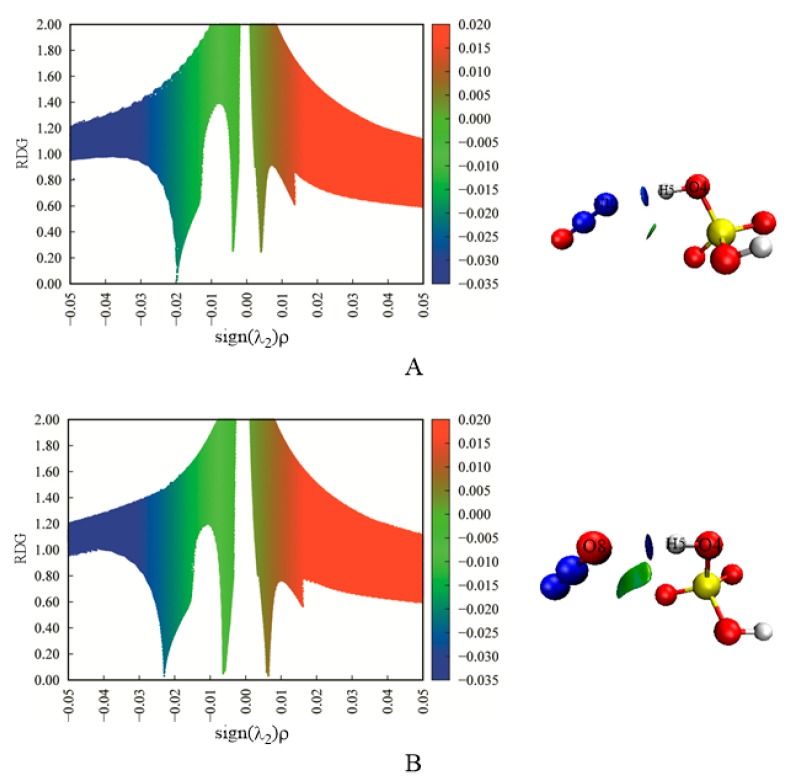
The scatter diagrams (left) and isosurfaces (right) of RDG analyses for complexes (**A**) and (**B**). The blue, green, and red colors represent the H-bond interactions, Van der Waals interactions, and steric effects, respectively.

To further verify the formation of intermolecular H-bonds mentioned above, we have performed RDG analyses for the complexes A and B. As shown in Figure 2, the intermolecular H-bonds can be verified by the presence of the corresponding spikes between H_2_SO_4_ and N_2_O.

**Figure 3:**
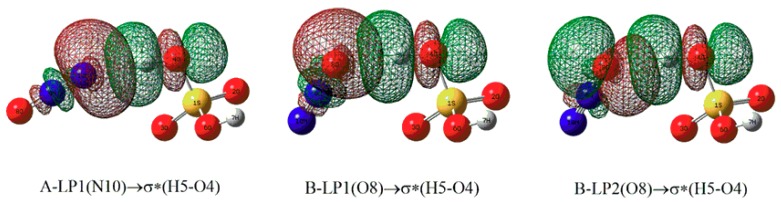
The NBO orbital interaction diagrams for the H-bonds in complexes A and B, where the isodensity contours are 0.001 electron/bohr^3^.

To explore the formation mechanisms for the above intermolecular H-bonds, NBO analyses have been carried out based on the optimized complexes. As shown in Figure 3, the formation of the intermolecular H-bonds in complexes A and B is mainly attributed to the electron transfer from the lone pairs of the terminal N and O atoms of N_2_O to the anti-bonding σ orbital (σ*) of the O4-H5 bond of H_2_SO_4_, respectively. Moreover, as given in Table 2, the larger total second-order stabilization energies of the orbital interactions in complex B imply that the H-bond in complex B is stronger than that of complex A, which can be further confirmed by the interaction energy mentioned below.

**Figure 4:**
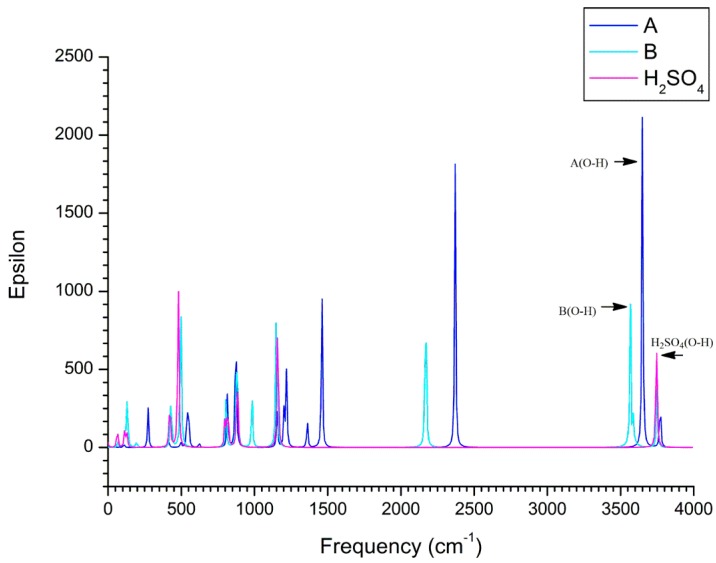
Calculated IR spectra of complexes A and B as well as H_2_SO_4_.

According to the NBO analyses, the O4-H5 bond of H_2_SO_4_ participating in the H-bonds should be elongated due to the electron transfer from the lone pairs of the proton acceptor to the σ* orbital of the proton donor. As a result, compared with the isolated state, the O4-H5 bond of H_2_SO_4_ has been elongated by about 0.006 and 0.008 A, respectively. Correspondingly, as shown in Figure 4, red-shifts of 115 and 152 cm^−1^ occur for the vibrational frequencies of the O4-H5 bond in complexes A and B, respectively. Moreover, the characteristic absorption peaks at 3650.0 and 3612.8 cm^−^^1^ are originated from the stretching vibration of the O4-H5 bond of H_2_SO_4_ fragment in complexes A and B, providing the direct spectra proof for the existence of intermolecular H-bonds. For the sake of reference, the detailed IR results including peak positions and intensities have been given in Table S1 of the Supplementary Material (SM).

**Figure 5:**
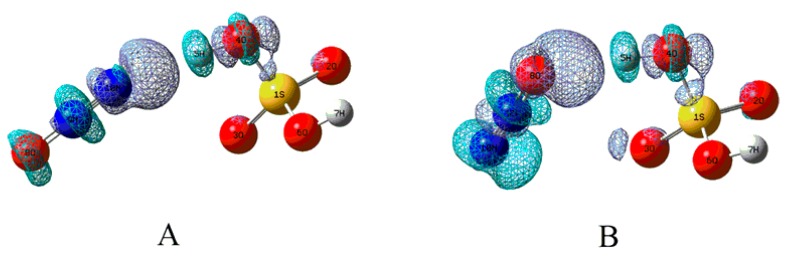
Electron density difference maps for complexes (**A**) and (**B**). Blue and violet areas denote the loss and gain of electron density, respectively. The isodensity contours are 0.001 electron/bohr^3^.

To investigate the electron redistribution details for H_2_SO_4_ and N_2_O upon complexation, the electron density difference maps of them have been constructed based on the optimized complexes A and B. As shown in Figure 5, electron redistribution mainly occurs along the intermolecular H-bond axis in the complexes, which is consistent with the features of the H-bonding complexes [39,40,41]. In more details, in complex A, the loss of the electron is mainly concentrated on the positions of the proton involved in the intermolecular H-bond in H_2_SO_4_ and the other two atoms of N_2_O not participating in the H-bonds. As for the gain of the electron, it is mainly concentrated on the terminal N atom of N_2_O and the O atom of H_2_SO_4_ involving in the H-bonds. Similarly, the same is also true for complex B. As a result, it was found that N_2_O and H_2_SO_4_ act as an electron donor and electron acceptor in both complexes, where the net electron transfer is 0.014 and 0.017 in complexes A and B based on the NBO analyses, respectively.

#### 3.1.2. Energy Analyses

As displayed in Table 3, the calculated interaction energies between H_2_SO_4_ and N_2_O are −2.14(−2.72) and −2.77(−4.22) kcal/mol in complexes A and B, respectively, where the data in parentheses refer to the results at the CCSD(T)/AUG-cc-pVTZ level of theory. As a result, complex B is more stable than complex A by 0.76 (1.72) kcal/mol, which is in agreement with the larger H-bond in complex B.

**Figure 6:**
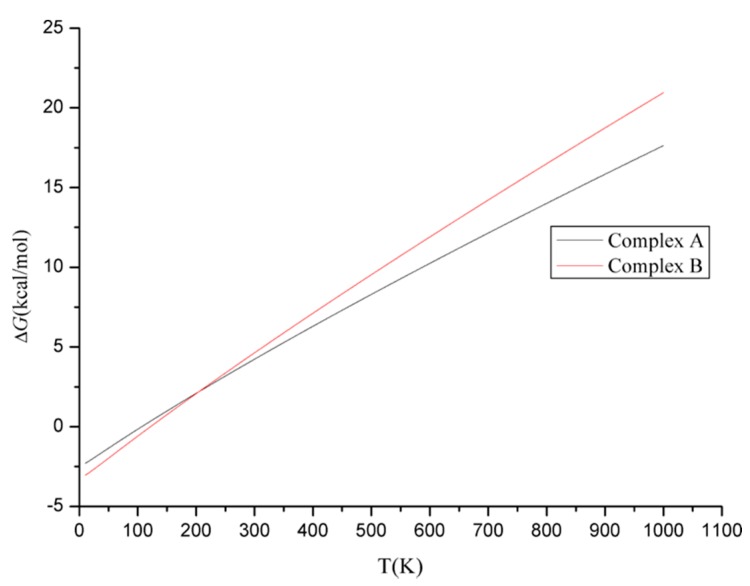
The dependence of Δ*G* on temperatures in the formation process of complexes A and B.

Thermodynamically, the calculated enthalpy changes (Δ*H*) for complexes A and B are all negative, indicating that the formation processes of them are exothermic reactions. On the other hand, the small positive values for the Gibbs free energy changes (Δ*G*) suggest that the formation processes of them are non-spontaneous at 298 K. As shown in Figure 6, the Δ*G* values become more negative with the decreasing of the temperature. Therefore, low temperature should be favorable for the formation of complexes thermodynamically.

#### 3.1.3. Molecular Dynamics Analyses

**Figure 7:**
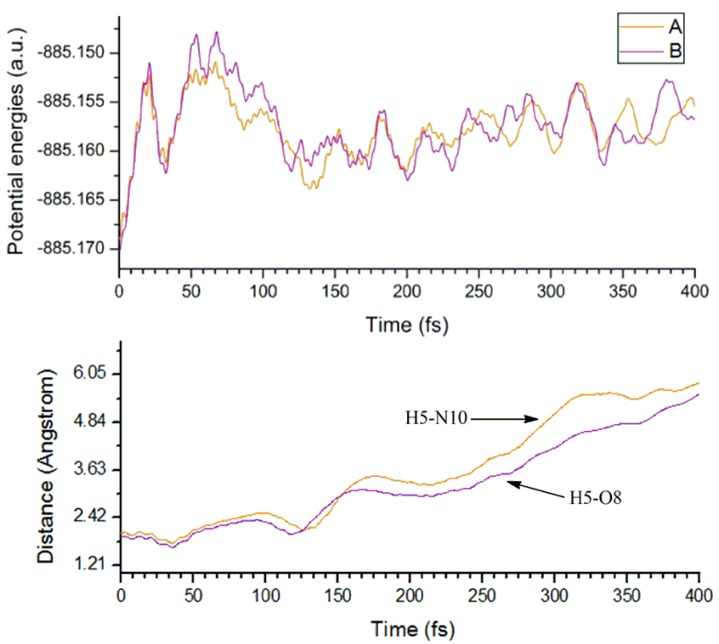
The time evolution of the potential energy (**top**) and the intermolecular H-bonds in complexes A and B (**bottom**) in molecular dynamics calculations.

To further explore the dynamic stability of the complexes A and B, molecular dynamics simulations have been performed on the basis of their optimized geometries. The time evolution of the potential energy and the H-bond in both complexes have been given in Figure 7. On the one hand, both intermolecular H-bonds in complexes can still be observed during the initial stage of the dynamics simulation, indicating that the complexes can exist in a short period of time. On the other hand, the intermolecular H-bonds disappear with the evolution of the simulation time, implying the instability of the complexes. Therefore, complexes A and B can exist transiently within a certain period of time.

### 3.2. Electron Capture Process

On the basis of the neutral complexes A and B mentioned above, the electron capture properties of them have been investigated. 

#### 3.2.1. Structural Features for the Electron Capture Products

**Figure 8:**
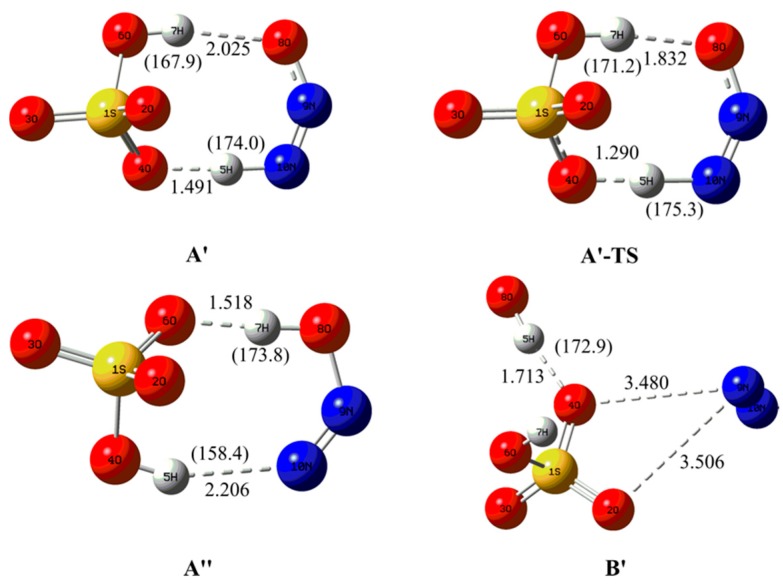
The optimized electron capture products and the related transition state.

**Figure 9:**
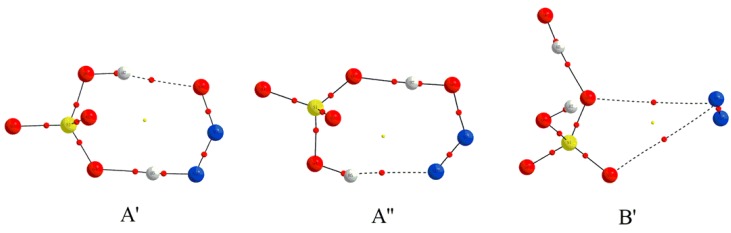
The molecular graphs for the electron capture products, where the BCP and ring critical point are denoted as small red and yellow dots, respectively.

**Figure 10:**
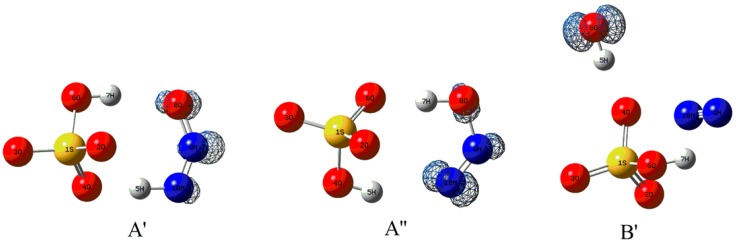
Spin density distribution diagram of the electron capture products, where the isodensity contours are 0.03 electron/bohr^3^.

As shown in Figure 8, complex A′ has been located for the complex A upon electron capture. Obviously, the original H5 atom of H_2_SO_4_ fragment has been transferred to the terminal N10 atom of N_2_O, resulting in the formation of two fragments HSO_4_^−^ and HNNO as well as a new intermolecular H-bond N10-H5···O4. At the same time, an additional intermolecular H-bond O6-H7···O8 has also formed between the other proton of H_2_SO_4_ and the terminal O8 atom of N_2_O. As shown in Figure 9, complex A′ is characterized by the double intermolecular H-bonds from the presence of the BCPs and the spikes between two fragments in RDG maps as displayed in Figure S1 of Supplementary Materials. Especially, for the intermolecular H-bond N10-H5···O4, its larger strength should be highlighted from its short H-bonding distance of 1.491 A. Actually, as presented in Table 1, the positive and negative values of the Laplacian and energy density at the BCP of H-bond suggest that it has partial covalent properties. In this case, can H atom transfer occur for the H-bond N10-H5···O4? To clarify this point, the H atom transfer behavior has been studied for complex A′. As a result, it was found that another complex A′′ can be obtained via the double H atom transfer, i.e., the H5 atom has been transferred from N10 of N_2_O to O4 of H_2_SO_4_ accompanying with the transfer of H7 from O6 of H_2_SO_4_ to O8 of N_2_O, where the corresponding transition state A′-TS has been confirmed by the IRC calculations. Similar to complex A′, as shown in Figure 9 and Figure S1 of Supplementary Materials, complex A′′ is also characterized by the double intermolecular H-bonds. Moreover, analyses of the energy parameters suggest that the forward and reverse barrier heights are 0.31(−1.44) and 9.05(7.62) kcal/mol, where the data in parentheses are the results including the ZPVE corrections. Clearly, the disappearance of the forward barrier height suggests that the reaction can take place readily. Meanwhile, the calculated ∆*H* and ∆*G* for the conversion process from complex A′ to A′′ are −8.74 and −9.39 kcal/mol, implying that the conversion process is also favorable thermodynamically. Therefore, complex A should be converted to complex A′′ upon electron capture, where the dynamic stability of the complex A′′ will be explored below. In addition, as displayed in Figure 10, the excess electron has been mainly distributed on the HNNO and NNOH fragments, exhibiting the nature of them as a radical.

For complex B, as displayed in Figure 8, its original intermolecular H-bond has disappeared upon electron capture and the whole complex has been collapsed into three fragments including OH radical, N_2_, and HSO_4_^−^. As displayed in Figure 10, the excess electron is mainly localized on the OH fragment. As a result, OH radical can be produced in the electron capture process of complex B. Therefore, besides the main source of the OH production (e.g., solar radiation), the present study can also provide an alternative source of the OH radical in the atmosphere although its contribution to the total OH concentration is unknown at present.

In addition, to verify the fact that the above electron capture products are free from the overestimated stabilization of the delocalized state for B3LYP method, relevant calculations have also been carried out employing the long range corrected functional CAM-B3LYP method and the 6-311++G(3df,3pd) basis set. As a result, the calculated results are well consistent with the B3LYP results.

#### 3.2.2. Electron Affinity

To explore the ability of the neutral complexes A and B to accept an electron thermodynamically, the electron affinities of them have been calculated. As shown in Table 4, the AEAs are 1.80 and 3.18 eV for complexes A and B, respectively. Here, the small AEA of complex A relative to complex B should be attributed to the instability of the electron capture product A′ mentioned above. The positive values of AEA suggest that the electron capture products are more stable than those of the neutral complexes. At the same time, the large difference between AEA and VEA suggests the significant geometrical changes for complexes A and B before and after electron capture. Obviously, this point is consistent with the optimized geometries for complexes A′ and B′ mentioned above. In addition, the calculated VEDEs of the complexes A′ and B′ are 5.22 and 7.44 eV, respectively. Therefore, the electron capture products are stable relative to the detachment of an electron. 

In addition, the ∆*G* for the electron capture process of complexes A and B are −38.9 and −80.2 kcal/mol, respectively. Therefore, the electron capture processes for the neutral complexes are exothermic and spontaneous processes and can occur easily thermodynamically.

#### 3.2.3. Electron Capture Dynamics

To further explore the microscopic details in the electron capture process, the molecular dynamics calculations have been carried out based on the optimized complexes A and B.

**Figure 11:**
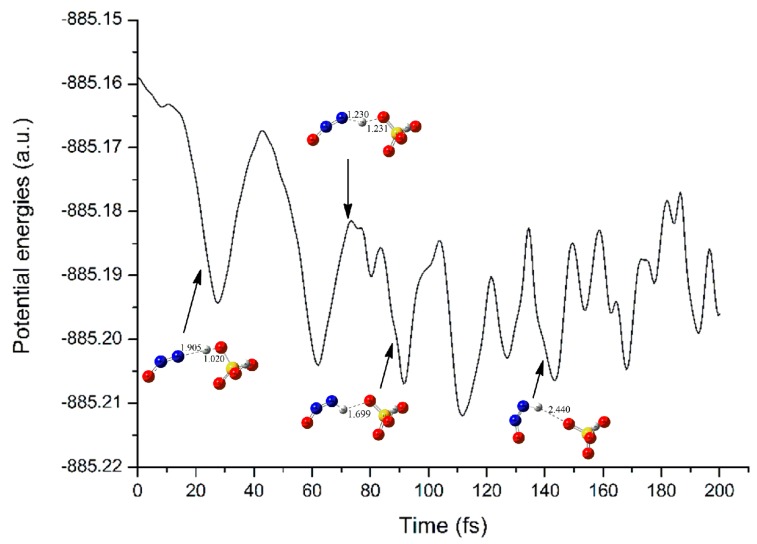
The changes of potential energy and main geometries in the electron capture process of complex A along with the simulation time.

As shown in Figure 11 and Figure S2 of Supplementary Materials, for complex A upon electron capture, H_2_SO_4_ and N_2_O begin to approach to each other, accompanying with the strengthening of the intermolecular H-bond. Meanwhile, the O4-H5 bond participating in the H-bond in H_2_SO_4_ fragment increases gradually. On the contrary, the distance between the terminal N10 atom of N_2_O and the H5 atom of H_2_SO_4_ fragment decreases simultaneously. As a result, the H5 atom of H_2_SO_4_ fragment has been transferred to the terminal N10 atom of N_2_O fragment. For example, at 88.5 fs, the distances N10···H5 and O4···H5 have been changed from the initial 2.030 and 0.974 A to 0.981 and 1.699 A, respectively. At 134.5 fs, the H5 atom of H_2_SO_4_ has been transferred to the N10 atom of N_2_O, producing the HNNO and HSO_4_^−^ fragments, which is consistent with the optimized complex A′.

**Figure 12:**
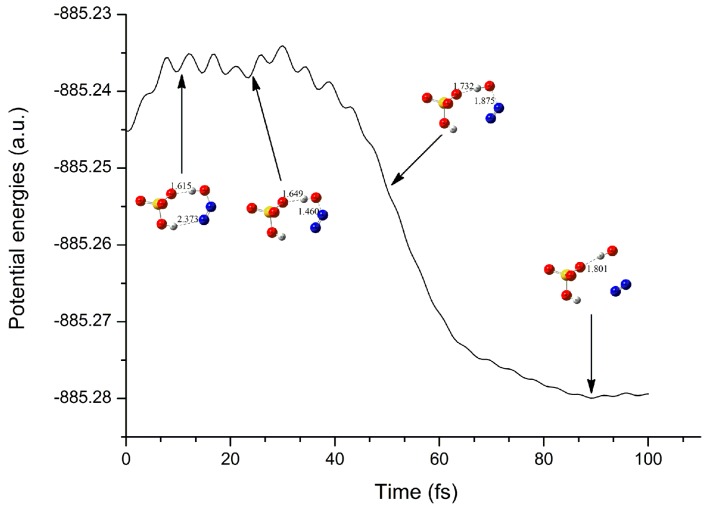
The changes of potential energy and main geometries for complex A′′ along with the simulation time.

Moreover, as mentioned above, complex A′ can be transformed into complex A′′ easily. To further explore the dynamic stability of complex A′′, molecular dynamics calculations have been carried out based on its optimized geometry. As displayed in Figure 12 and Figure S3 of Supplementary Materials, the N-O bond of HONN fragment has been elongated gradually with the evolution of simulation time. At 60 and 90 fs, the N-O bond has been elongated to 2.128 and 2.950 A, respectively. Therefore, complex A′′ is unstable and can be further transformed into OH radical, N_2_, and HSO_4_^−^ fragments.

**Figure 13:**
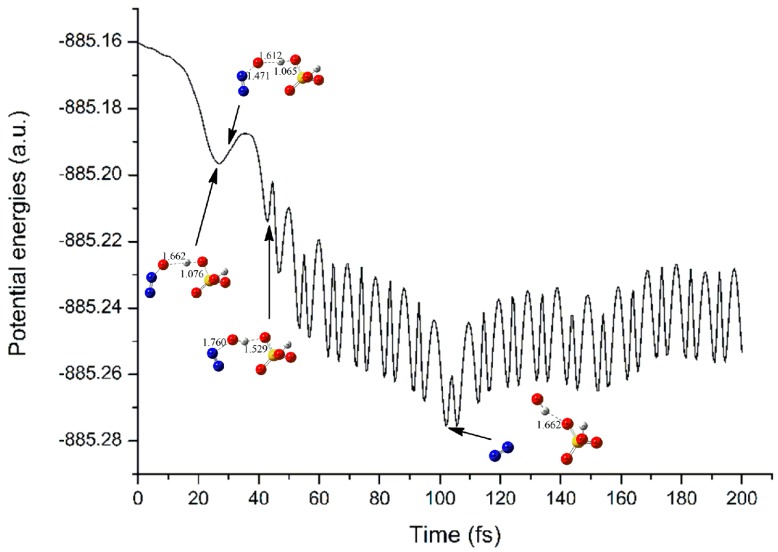
The changes of potential energy and main geometries in the electron capture process of complex B along with the simulation time.

As for the complex B upon electron capture, as shown in Figure 13 and Figure S4 of Supplementary Materials, the H5 atom involved in the intermolecular H-bond in H_2_SO_4_ fragment approaches the terminal O8 atom of N_2_O fragment gradually, accompanying with the elongation of the N9-O8 bond of N_2_O fragment simultaneously. At 50 fs, the O4-H5 bond of H_2_SO_4_ and N9-O8 bond of N_2_O have been changed from 0.976 and 1.191 to 1.473 and 2.047 A, respectively. With the evolution of the simulation time, complex B has been transformed into OH radical, N_2_, and HSO_4_^−^ fragments, which is consistent with the optimized geometry of complex B′.

In addition, as shown in Figure 11 and Figure 13, the energies of the neutral complexes have been decreased significantly upon electron capture, reflecting the easy proceeding of the electron capture process once again.

As a result, the neutral complexes A and B can be eventually transformed into OH radical, N_2_, and HSO_4_^−^ fragments upon electron capture. Certainly, further experiments are needed to further confirm the present findings.

## 4. Conclusions

In the present study, the electron capture properties of the formed complex of H_2_SO_4_ with N_2_O in the gas phase have been systematically explored theoretically. It was found that H_2_SO_4_ and N_2_O can form two transient complexes via intermolecular H-bonds in the atmosphere, which has been characterized by using AIM, NBO, and RDG analyses. Upon electron capture, two neutral complexes have been eventually transformed into OH radical, N_2_, and HSO_4_^−^ species. Therefore, the present study not only provides an alternative formation source of OH radical in the atmosphere as well as new insights into the degradation and transformation behavior of N_2_O, but also enables us to better understand the potential role of H_2_SO_4_ and the excess electron in the atmosphere. Certainly, more extensive experiments are highly desirable to verify the present findings.

## Figures and Tables

**Table 1:** The topological parameters at the BCPs for the intermolecular H-bonds *^a^*.

Complexes	BCP	*ρ*_bcp_	∇^2^*ρ*_bcp_	*G*_bcp_	*V*_bcp_	*H*_bcp_
A	O4-H5···N10	0.0198	0.0694	0.0155	−0.0137	0.0018
B	O4-H5···O8	0.0230	0.0813	0.0190	−0.0177	0.0013
A′	O6-H7···O8	0.0201	0.0681	0.0156	−0.0142	0.0014
	N10-H5···O4	0.0816	0.0897	0.0550	−0.0876	−0.0326
A″	O8-H7···O6	0.0727	0.1029	0.0523	−0.0788	−0.0266
	O4-H5···N10	0.0147	0.0494	0.0103	−0.0083	0.0020
B′	O8-H5···O4	0.0424	0.1090	0.0340	−0.0408	−0.0068

*^a^* Atomic numbering refers to Figure 1 and Figure 8.

**Table 2:** NBO analyses for the H-bonds in complexes A and B *^a^*.

Complexes	Donor NBO	Acceptor NBO	*E*^(2)^
A	LP(N10)	σ*(H5-O4)	6.38
B	LP1(O8)	σ*(H5-O4)	2.91
	LP2(O8)	σ*(H5-O4)	4.79

*^a^* The unit of *E*^(2)^ is in kcal/mol.

**Table 3:** The calculated interaction energies (Δ*E*_Int_), relative energies (Δ*E*_Rel_), enthalpy and Gibbs free energy changes (Δ*H* and Δ*G*) during the formation process of complexes A and B *^a^*.

Complexes	Δ*E*_Int_	Δ*E*_Rel_	Δ*H*	Δ*G*
A	−2.14(−2.72)	0.76(1.72)	−2.07(−2.34)	4.19(3.92)
B	−2.77(−4.22)	0.00(0.00)	−2.92(−3.93)	4.58(3.57)

*^a^* The data in parentheses refer to the results at the CCSD(T)/AUG-cc-pVTZ level of theory plus the thermodynamic corrections at the B3LYP/6-311++G(3df,3pd) level of theory. All the units are in kcal/mol.

**Table 4:** AEA, VEA, and VEDE results for complexes A and B *^a^*.

Complexes	AEA	VEA	VEDE
A	1.80	−0.27	5.22
B	3.18	−0.27	7.44

*^a^* All the units are in eV.

## Supplementary Materials

RDG maps of the electron capture products, the time evolution of the selected bond distances for complexes A, A′′, and B in the molecular dynamics calculations, and the IR data for complexes A and B. The following are available online, Figure S1: RDG maps of the electron capture products, Figure S2: The evolution of the selected bond distances for complex A in the electron capture process as a function of time, Figure S3: The evolution of the selected bond distances for complex A′′ as a function of time, Figure S4: The evolution of the selected bond distances for complex B in the electron capture process as a function of time, Table S1: The calculated IR results for complexes A and B.
