# Supplementary material for: Theoretical Insights into the Electron Capture Behavior of H2SO4···N2O Complex: A DFT and Molecular Dynamics Study
Source: Molecules. 2018 Sep 13;23(9):2349. doi: 10.3390/molecules23092349 (PMC6225230; doi:10.3390/molecules23092349)
Supplement: Supplementary file 1 [file molecules-23-02349-s001.pdf]

Theoretical insights into the electron capture behavior of $H_2SO_4\cdots N_2O$ complex: A DFT and molecular dynamics study

Weihua Wang[a,*], Wenling Feng[a], Wenliang Wang[a], Ping Li[a,*]
[a] Key Laboratory of Life-Organic Analysis, School of Chemistry and Chemical Engineering, Qufu Normal University, Qufu, 273165, P. R. China

E-mails: wwh78@163.com (Weihua Wang) and lignip@163.com (Ping Li)

Supporting Information

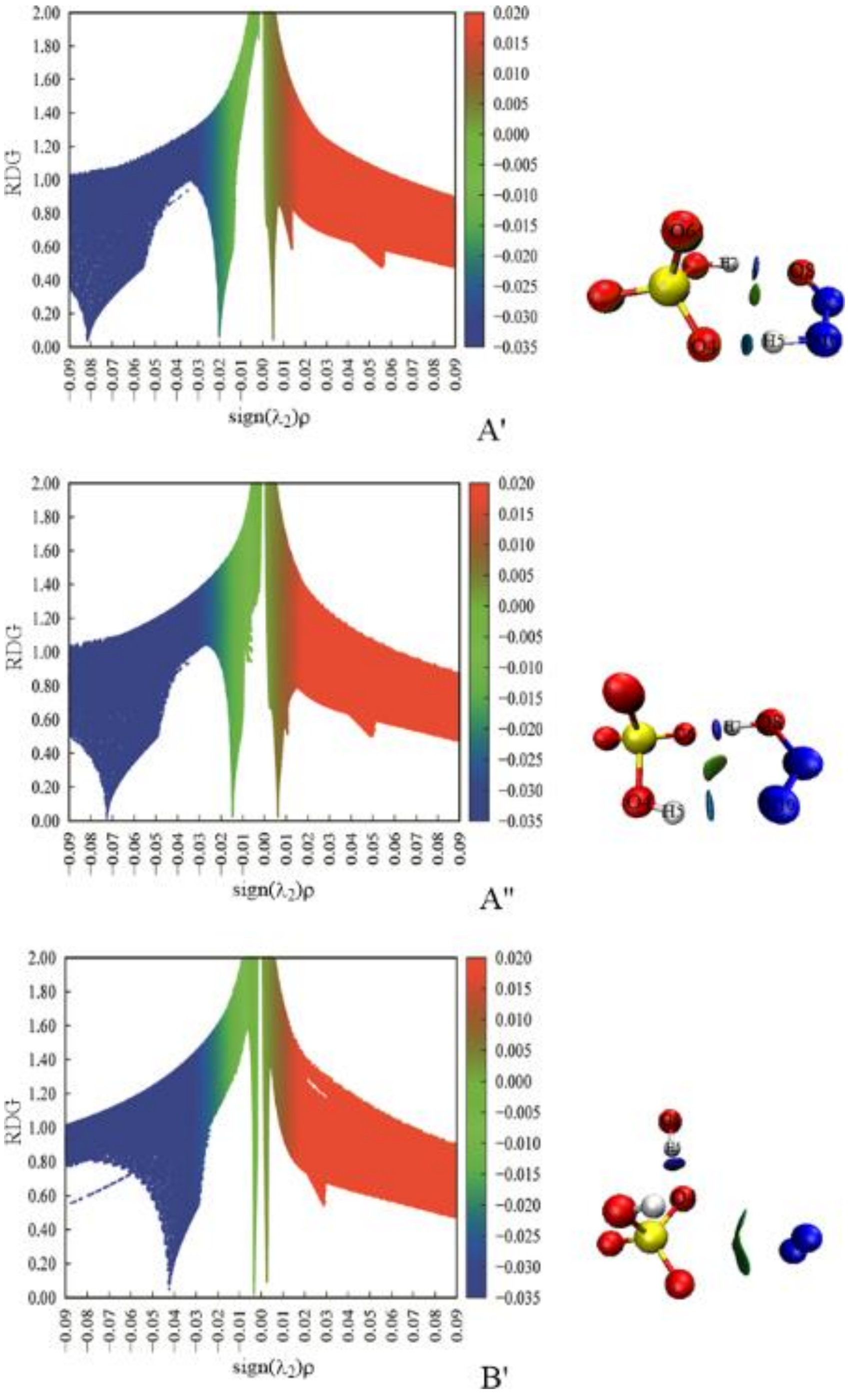

Figure S1. RDG maps of the electron capture products.

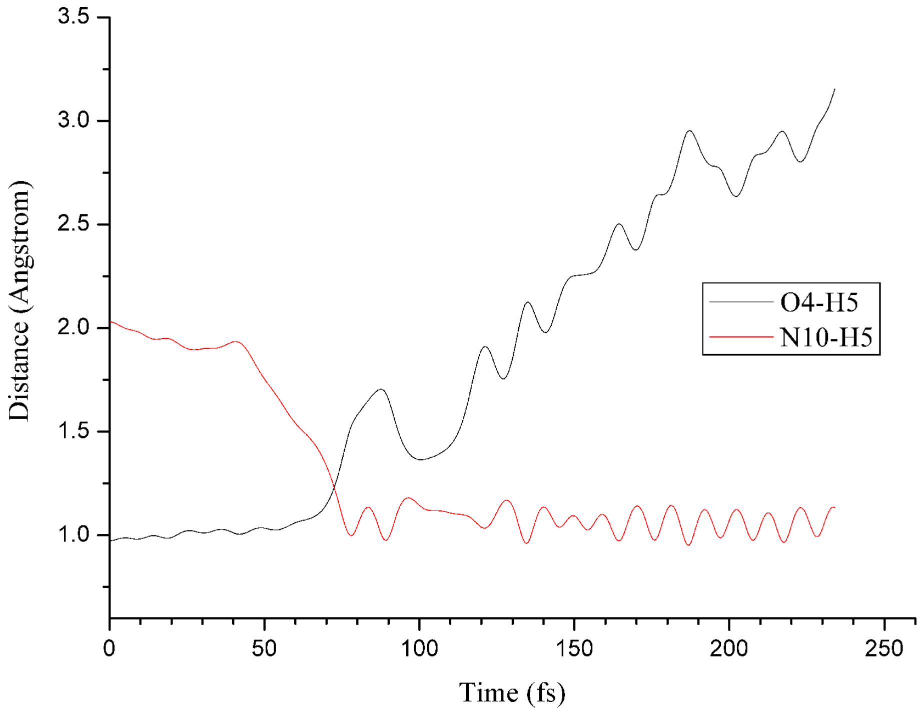

Figure S2. The evolution of the selected bond distances for complex A in the electron capture process as a function of time.

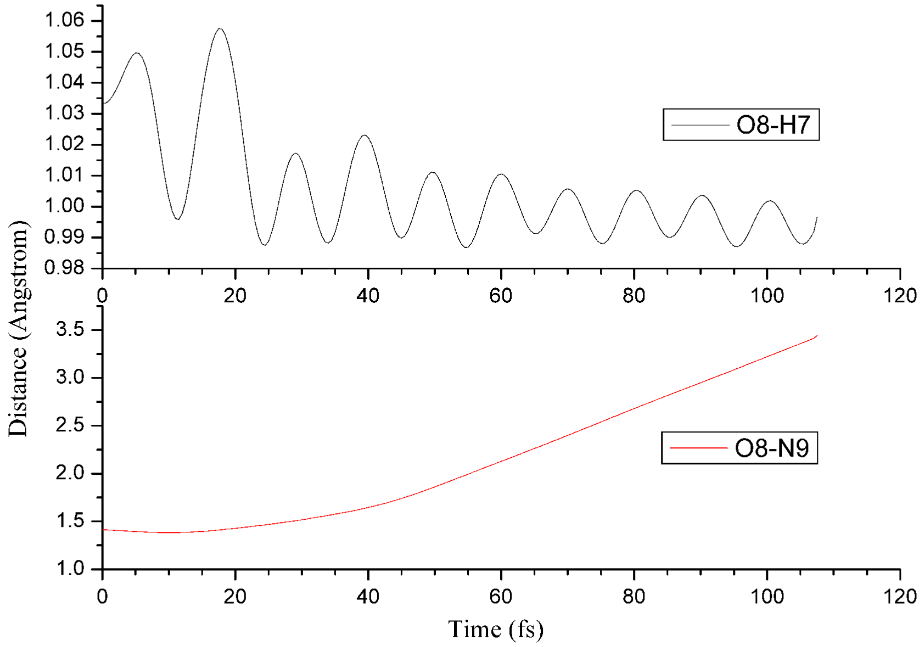

Figure S3. The evolution of the selected bond distances for complex A′′ as a function of time.

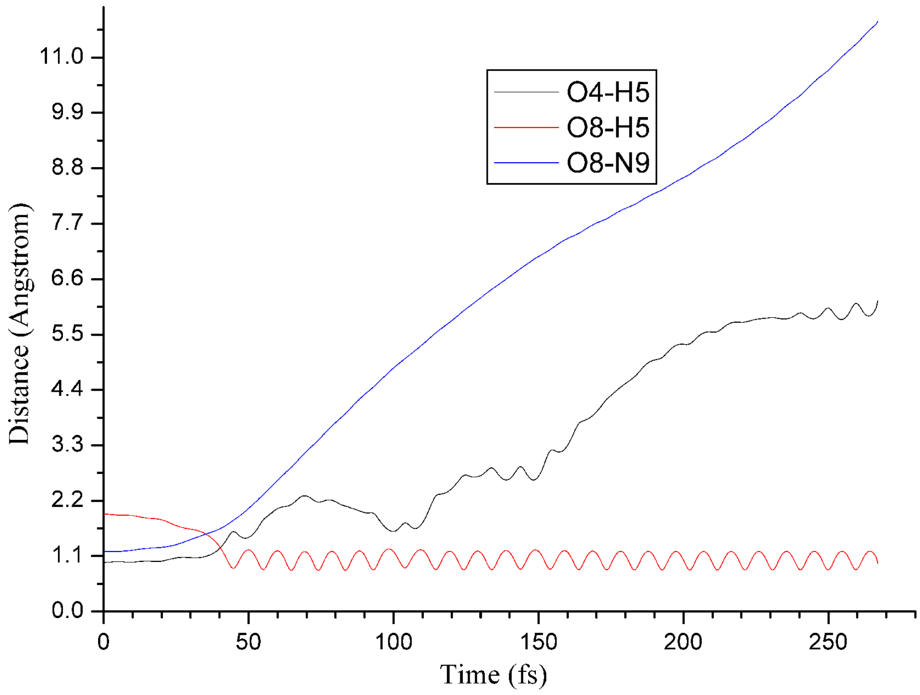

Figure S4. The evolution of the selected bond distances for complex B in the electron capture process as a function of time.

Table S1. The calculated IR results for complexes A and B

Complex A		Complex B	
Frequencies (cm^{-1})	IR Intensities (KM/Mole)	Frequencies (cm^{-1})	IR Intensities (KM/Mole)
11.79	0.93	24.38	1.28
21.26	1.43	41.36	1.99
51.83	1.57	52.89	1.82
79.54	1.48	103.94	5.85
108.44	8.07	140.91	5.35
273.35	75.30	266.44	81.01
372.97	1.90	371.01	1.41
411.84	22.95	413.13	22.38
496.13	12.43	496.73	9.76
534.33	27.74	536.14	24.17
546.88	86.30	550.25	84.51
553.10	20.38	556.06	22.78
621.08	5.10	603.70	3.88
622.18	5.64	611.13	9.67
815.77	116.76	818.77	111.68
872.91	323.59	877.88	310.99
1153.99	82.00	1152.08	87.90
1203.41	96.09	1215.02	129.53
1219.19	139.40	1224.45	91.73
1360.64	59.50	1310.41	99.48
1460.40	319.50	1458.59	327.49
2370.50	533.37	2364.52	343.67
3650.00	650.21	3612.78	592.85
3770.84	115.83	3769.09	120.16
